# PET Imaging of Synaptic Density: Challenges and Opportunities of Synaptic Vesicle Glycoprotein 2A PET in Small Animal Imaging

**DOI:** 10.3389/fnins.2022.787404

**Published:** 2022-03-08

**Authors:** Takuya Toyonaga, Arman Fesharaki-Zadeh, Stephen M. Strittmatter, Richard E. Carson, Zhengxin Cai

**Affiliations:** ^1^Positron Emission Tomography (PET) Center, Radiology and Biomedical Imaging, Yale School of Medicine, New Haven, CT, United States; ^2^Psychiatry, Yale School of Medicine, New Haven, CT, United States; ^3^Neurology, Yale School of Medicine, New Haven, CT, United States; ^4^Neuroscience, Yale School of Medicine, New Haven, CT, United States

**Keywords:** SV2A, brain PET, small animal, synapse, neurodegeneration

## Abstract

The development of novel PET imaging agents for synaptic vesicle glycoprotein 2A (SV2A) allowed for the *in vivo* detection of synaptic density changes, which are correlated with the progression and severity of a variety of neuropsychiatric diseases. While multiple ongoing clinical investigations using SV2A PET are expanding its applications rapidly, preclinical SV2A PET imaging in animal models is an integral component of the translation research and provides supporting and complementary information. Herein, we overview preclinical SV2A PET studies in animal models of neurodegenerative disorders and discuss the opportunities and practical challenges in small animal SV2A PET imaging. At the Yale PET Center, we have conducted SV2A PET imaging studies in animal models of multiple diseases and longitudinal SV2A PET allowed us to evaluate synaptic density dynamics in the brains of disease animal models and to assess pharmacological effects of novel interventions. In this article, we discuss key considerations when designing preclinical SV2A PET imaging studies and strategies for data analysis. Specifically, we compare the brain imaging characteristics of available SV2A tracers, i.e., [^11^C]UCB-J, [^18^F]SynVesT-1, [^18^F]SynVesT-2, and [^18^F]SDM-16, in rodent brains. We also discuss the limited spatial resolution of PET scanners for small brains and challenges of kinetic modeling. We then compare different injection routes and estimate the maximum throughput (i.e., number of animals) per radiotracer synthesis by taking into account the injectable volume for each injection method, injected mass, and radioactivity half-lives. In summary, this article provides a perspective for designing and analyzing SV2A PET imaging studies in small animals.

## Introduction

Synapses are the basic communication units for neurons. The disruption of synapse homeostasis has been observed in a variety of neuropsychiatric disorders (Lepeta et al., 2016). The number of synapses in the human cortex is estimated to be about 360 trillion (Roth and Dicke, 2005). Direct quantification of synapses has been realized by using stereotactic electron microscopy, confocal microscopy, and single photon imaging at the microscopic level. Positron emission tomography (PET) imaging using protein-specific imaging probes has allowed for the *in vivo* quantification of many proteins targets in the brain (Gunn et al., 2015). We have recently been able to map and quantify the synaptic vesicle glycoprotein 2A (SV2A) in the living brain, with the emergence of SV2A-specific imaging probes. Since SV2A can be found in glutamatergic and GABAergic neurons, it holds great promise as a general biomarker of synaptic density (Bajjalieh et al., 1994). Great efforts have been devoted to the clinical and preclinical applications of SV2A PET in a variety of neuropsychiatric disorders. While numerous clinical SV2A PET studies are ongoing in expanding the applications of SV2A PET in the study of more diseases and physiological processes, preclinical SV2A PET studies using small animal models of neuropsychiatric disorders are important, e.g., in evaluating experimental drug treatment effects longitudinally. In this article, we will focus on (1) the opportunities and challenges of SV2A PET in small animal brain PET and (2) ways to strengthen the robustness of preclinical SV2A PET studies through careful experimental design and proper data analysis strategies.

### Applications of SV2A PET in Clinical Neuropsychiatric Disorders

Since the initial publications on SV2A PET imaging using [^18^F]UCB-H (Warnock et al., 2014), followed by [^11^C]UCB-J (Finnema et al., 2016; Nabulsi et al., 2016), which has higher specific uptake, SV2A PET has been used in human studies in a variety of neuropsychiatric disorders (Cai et al., 2019; Finnema et al., 2021).

The first patient population SV2A PET was applied to was temporal lobe epilepsy (TLE) patients and they discovered the high binding potential (*BP*_ND_) asymmetry indices in the hippocampi of TLE patients (Finnema et al., 2016, 2020). SV2A PET has also been used in multiple clinical studies in Alzheimer’s disease (AD) patients. The pioneering studies reported by Chen et al. (2018) and Mecca et al. (2020) revealed 25–27% decreased SV2A density in the hippocampus of AD patients. In a pilot SV2A PET study in Parkinson’s disease patients and healthy control subjects, lower *BP*_ND_ was observed in substantia nigra and other gray matter regions (Matuskey et al., 2020). In other studies, patients with high severity depression, schizophrenia, and cannabis use disorder showed significantly lower tracer uptake in the primary regions (Holmes et al., 2019; D’Souza et al., 2020; Onwordi et al., 2020). Based on these promising clinical imaging data, we anticipate more follow-up and larger scale investigations, as well as the use of SV2A PET in evaluating therapeutic effects in clinical trials.

While human studies have been conducted on many clinical diseases, preclinical investigations using the corresponding disease animal models are informative to validate the clinical imaging findings.

### Animal Models in Neuroscience

Rodents are robust species for neuroscience to model neuropsychiatric diseases and to evaluate, for example, treatment effects, drug toxicity, behavior, and disease phenotypes. From the 1970s to 1980s, the most common experimental rodent was rat (approximately, rat: mouse = 4:1 in publication number) (Ellenbroek and Youn, 2016). However, gene manipulation techniques became available for mouse (Thomas and Capecchi, 1987), much earlier than in the rat (Geurts et al., 2009), substantially boosting mouse research. Although more transgenic disease models are available in mouse, the rat has several advantages: (1) ease of handling, (2) less invasiveness of surgery, and (3) more variety of behavioral tests. If there were appropriate rat disease models, the larger brain size is also very helpful to compensate for the limited PET image resolution (details in the following section).

### Applications of Synaptic Vesicle Glycoprotein 2A ([^11^C]UCB-J) PET Using Small Animal Models

Several neuropsychiatric disease models have been reported using SV2A tracers with cross-sectional or longitudinal study designs. For example, transgenic AD mice (APP/PS1) showed 26.2% lower standardized uptake value ratio minus one using brain stem as a reference region (SUVR-1_BS_) at 30–60 min in hippocampus than wild-type (WT) mice using [^11^C]UCB-J (Toyonaga et al., 2019). Other SV2A PET studies in mouse models with different gene manipulations also showed significant SV2A density decreases in primary regions as follows. In the heterozygous Thy1-αSyn mouse model for Parkinson’s disease (PD), 12% lower area under the curves ratio between brain regions to blood was seen in hippocampus from 0 to 60 min [^11^C]UCB-J data (Xiong et al., 2021). In the homozygous SAP90/PSD-95-associated protein 3 (Sapap3) knockout mouse model for obsessive-compulsive disorder, 14% lower volume of distribution (*V*_T_) using image-derived input function (IDIF) was observed in striatum based on the Logan reference method (Glorie et al., 2020). The heterozygous Q175DN knock-in mice for Huntington’s disease (HD) demonstrated 20% lower *V*_T(IDIF)_ in striatum (Bertoglio et al., 2021). For rat SV2A PET studies, Thomsen et al. (2021) generated PD and HD lesion by the local injections of 6-OHDA and quinolinic acid (QA), respectively, in the rat striatum. PD lesion showed 6.2% *V*_T(IDIF)_ decrease, and HD lesion showed QA dose-dependent decrease (39.3% for 20μg and 55.1% for 40μg). Raval et al. (2021) showed 8.9% *V*_T(IDIF)_ decrease in striatum by the 6-hydroxydopamine (6-OHDA) local injection in the medial forebrain bundle and rostral substantia nigra. Their results suggested that the striatal synaptic loss was induced by the loss of projecting synapses from dopamine neuron. The systemic kainic acid injection was conducted to establish a TLE rat model and on average 22% decrease of SUV at 20–40 min was found in the six different brain regions (Serrano et al., 2020). In terms of study design, longitudinal evaluation is one of the biggest advantage of the *in vivo* measurements, since repeated measures in the same animals improve the statistical power (Toyonaga et al., 2019). Currently, we are expanding the application of SV2A PET to many different animal models including stroke, aging, and spinal cord injury.

### Choice of Synaptic Vesicle Glycoprotein 2A PET Tracers

When scan protocols are planned, selection of PET tracers is an important factor. We have several options for SV2A tracers labeled with either ^11^C or ^18^F. With similar tracer kinetics (e.g., [^11^C]UCB-J and [^18^F]SynVesT-1), the tracer with the longer half-life would be advantageous in terms of scan throughput. For ^11^C tracers, only one set of dynamic scans can be conducted per radiosynthesis, thus animal throughput will be tied to the number of available scanners and the number of animals that can be scanned simultaneously per scanner. In contrast, ^18^F tracers can be used in multiple sequential scans per radiosynthesis.

**FIGURE 1:**
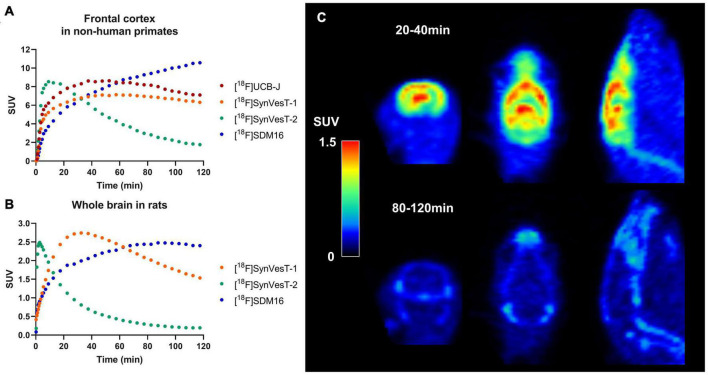
Tracer kinetics for ^18^F labeled SV2A tracers in rhesus macaques and rats. **(A)** Representative time activity curves (TACs) for [^18^F]UCB-J, [^18^F]SynVesT-1, [^18^F]SynVesT-2, and [^18^F]SDM-16 in frontal cortex of rhesus macaques from different subjects. **(B)** Representative TACs for [^18^F]SynVesT-1, [^18^F]SynVesT-2, and [^18^F]SDM-16 in whole brain of rats. **(C)** [^18^F]SynVesT-2 20–40 min (top) and 80–120 min (bottom) summed images in a wild-type Sprague–Dawley rat. The 20–40 min summed image shows high uptake in the brain while the 80–120 min image shows tracer uptake in skull and spine. The bone uptake at later time windows suggests defluorination.

There are several ^18^F-labeled SV2A tracers, i.e., [^18^F]UCB-H (Warnock et al., 2014), [^18^F]UCB-J (Li et al., 2019b), [^18^F]SynVesT-1 (Li et al., 2019a, 2021), [^18^F]SynVesT-2 (Cai et al., 2020b), and [^18^F]SDM-16 (Zheng et al., 2021), with different specific binding, brain kinetics, and peripheral metabolic rates. Figures 1A,B show the tracer kinetics in rhesus macaques and rats. Overall, tracer kinetics for [^18^F]SynVesT-2 is the fastest, and[^18^F]SDM-16 is the slowest for both species.

[^18^F]UCB-J was successfully synthesized and showed almost identical properties to [^11^C]UCB-J (Li et al., 2019b). However, the reported radiosynthesis method of [^18^F]UCB-J suffered from low radiochemical yield, which presents a major obstacle for small animal experiments unless alternative approaches with high radiochemical yields are developed. The relatively high non-specific brain uptake of [^18^F]UCB-H was found in rat, non-human primate and human brain PET imaging studies (Warnock et al., 2014; Bastin et al., 2020; Goutal et al., 2021). The second generation SV2A PET tracers ([^18^F]SynVesT-1, [^18^F]SynVesT-2, and [^18^F]SDM-16) possess higher specific binding in the brain. Of these tracers, [^18^F]SynVesT-1 is the most advanced and has been evaluated in rodents (Sadasivam et al., 2020), non-human primates (Li et al., 2019a), and healthy human subjects (Li et al., 2021; Naganawa et al., 2021).

We found that [^18^F]SynVesT-1 has excellent reliability in a rodent brain imaging study. The test-retest variability (equation below) of SUVR_BS_ at 30–60 min post-injection was −1.9 ± 4.2% (mean ± *SD*, *n* = 12) in hippocampus between two scans separated by about 4 weeks.


$$\mathrm{Test}-\mathrm{retest}\text{ variability}\left(\%\right)=200\times \frac{\text{test}{\mathrm{SUVR}}_{\text{BS}}-\text{retest}{\text{SUVR}}_{\text{BS}}}{\text{test}{\text{SUVR}}_{\text{BS}}+\text{retest}{\text{SUVR}}_{\text{BS}}}$$

Interestingly, [^18^F]SynVesT-2 showed *in vivo* defluorination at late time window (90–120 min post-injection) in rats (Figure 1C), but not in other species (Cai et al., 2020a,b). However, this is not likely to influence the quantitative analysis using this tracer, as [^18^F]SynVesT-2 has rapid brain kinetics and only requires ∼30 min scanning time for reliable quantitative analysis based on our previous non-human primates’ results (Cai et al., 2020b). [^18^F]SDM-16, a derivative of UCB-A (Estrada et al., 2016) is the most metabolically stable SV2A PET tracer so far (Zheng et al., 2021). Our evaluations of [^18^F]SDM-16 in rodents are ongoing and the results will be reported in due course. Generally speaking, tracers with slower brain kinetics need longer dynamic scans to generate quantitative data reliably, while shorter scan times are sufficient for tracers with faster kinetics. Also, tracers with different brain kinetics typically need different static imaging windows when SUVR is used instead of DVR/*BP*_ND_ as the outcome measurement.

## Discussion

### Challenges of Synaptic Vesicle Glycoprotein 2A PET in Small Animal Models

Small animal PET imaging has four major challenges, (1) scanner spatial resolution, (2) arterial blood sampling, (3) injection methods, and (4) molar activity and injection mass. We will discuss these issues in the following sections.

### PET Scanner

Even though PET scanners for small animals have much higher spatial resolution than clinical systems, the best resolution is still 1–1.5 mm full width at half maximum (FWHM) (Tai et al., 2005; Gaudin et al., 2021). Often this resolution is further degraded by smoothing which is part of the reconstruction algorithm. Since synaptic density imaging in neuropsychiatric disorders often evaluates regions with lower uptake than the other healthy regions, the partial volume effect (PVE) causes underestimation of the group difference due to spill-in from the surrounding higher uptake brain regions. Although it is impossible to avoid the PVE in small animal PET, using rats would reduce the impact of PVE, as rats have approximately three to four times larger brains than mice in volume (Badea et al., 2007; Welniak-Kaminska et al., 2019).

To compare the PVE difference between mouse and rat brains, the spatial resolution in rodent PET images was simulated using mouse and rat brain atlases as the ground truth (Ma et al., 2005; Mirrione et al., 2007; Papp et al., 2014). Gray matter regions in both atlases were filled with a value of 100 except for the hippocampus in which 100 was used for wild-type animals and 80 for AD rodents to simulate AD-like contrast, i.e., 20% lower SV2A density only in hippocampus. Then, a 3D Gaussian filter with 2 mm FWHM was applied to the simulated images, and mean values in neocortex and hippocampus were calculated by applying the original atlas regions. Based on the simulation, the 20% true AD: wild-type contrast was reduced to 11% in the rat and 8% in the mouse. If we assume a standard deviation of 10% of the mean, type I error (α) of 0.05 and type II error (β) of 0.20, power analysis shows the required sample sizes would be 14 per group for rats and 25 per group for mice, simply due to PVE. Therefore, using rats will increase the statistical power for PET studies and increase the likelihood of detecting significant differences in smaller brain regions, such as subregions of neocortex, subcortical nuclei, and spinal cord.

### Arterial Blood Sampling

Another practical challenge is arterial blood sampling in rodent imaging studies. Radioactivity counting and metabolite assay in arterial blood are necessary for kinetic analysis to produce gold standard results (Carson, 2003). However, catheterization of a major artery would be needed for blood sampling and the total volume of blood to be collected would be significant for small animals. One potential solution would be the use of an arteriovenous shunt with a dedicated detector pump system for continuous radioactivity counting (Mann et al., 2019), which involves several technical challenges, such as clogging and extracorporeal blood volume issues. One alternative solution for kinetic analysis is the image-driven input function (IDIF) method, such as how Bertoglio et al. (2020) measured *V*_T_ of [^11^C]UCB-J with a left ventricle IDIF. While this may be a very useful method, caveats of IDIFs include the PVE, cardiac motion artifact, and tracer uptake in the ventricle wall; these will affect the accuracy of the IDIF. A further critical point for both blood sampling and IDIF is radiometabolite assay. Multiple samples are needed to acquire the parent fraction time course; this can exceed the blood volume limit, especially for mice. Population-based metabolite corrections could be a solution, but these must be validated for each tracer, and ideally in each animal model.

Because of these challenges for estimating the input function, we have pursued kinetic analysis using reference tissue models. The kinetics of SV2A tracers, i.e., [^11^C]UCB-J and [^18^F]SynVesT-1, are known to be fit well with one-tissue compartment model based on the human and rhesus macaque data (Finnema et al., 2016; Nabulsi et al., 2016; Li et al., 2019a; Naganawa et al., 2021). For rodents, brain stem or cerebellum were used as a pseudo reference region to fit the other regional time activity curves (Toyonaga et al., 2019; Sadasivam et al., 2020). As comparison, the Logan graphical analysis in wild-type mice showed that there was linear fit even at t* = 0 (Sadasivam et al., 2020), which suggested that the tracer kinetics in rodent brain also can be described using one-tissue compartment model. A key assumption of reference region analyses is that there should be no group or time differences of the tracer uptake in the reference region, in order to avoid bias. This assumption is best if validated by ex vivo measurements, such as Western blotting or immunohistochemistry.

### Injection Methods

The injection method is also a key factor to conduct reliable PET imaging with high throughput. Although intravenous (IV) injection is the gold standard, it is sometimes challenging to accomplish genuine IV injection especially for mice and rats of small size or younger age. Small needles or catheters can be easily clogged or become dislodged from the vein during the injection process. In addition, the injection method should be simple enough to preclude introducing motion and to minimize the injection time discrepancy between animals if multiple animals are scanned on one scanner simultaneously.

**FIGURE 2:**
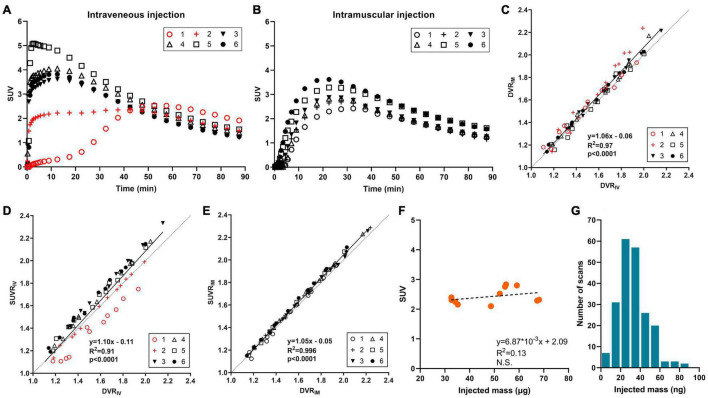
[^18^F]SynVesT-1 results in mice. **(A)** Time activity curves (TACs) with intravenous injection (2 animals with mixed intravenous/subcutaneous injection), and **(B)** TACs with intramuscular injection in six wild-type mice. **(C)** Correlation between distribution volume ratio (DVR) by intravenous injection (DVR_IV_) and DVR by intramuscular injection (DVR_IM_) for 12 brain regions. DVR was estimated with simplified reference tissue model (SRTM) using brain stem as a reference region. **(D)** Correlation between DVR_IV_ and standardized uptake value ratio by intravenous injection (SUVR_IV_) using 30–60 min post-injection data, also with brain stem normalization. **(E)** Correlation between DVR_IM_ and SUVR by intramuscular injection (SUVR_IM_). **(F)** Scatter plot of injected mass dose and whole brain SUV in wild-type mice (*n* = 11), and **(G)** histogram of SynVesT-1 injected mass dose in mice (*n* = 210).

To satisfy those requirements, intramuscular (IM) injection was evaluated by comparing the results acquired by IM and IV injection in the same animals. Six wild-type mice were scanned with [^18^F]SynVesT-1 on two separate days with IV and IM injections. Figure 2 shows the time activity curves (TACs) from the whole brain of each animal. The IV injection (Figure 2A) has two distinct outliers colored in red. Subject 1 had a much slower uptake than the others, which suggests that most of tracer was in the subcutaneous space. Subject 2 showed lower and flatter TACs in the early time frames, potentially due to mixed injections through IV and subcutaneous routes. In comparison, IM injections (Figure 2B) showed consistent TACs from all 6 animals. Then, distribution volume ratio (DVR) was calculated using brain stem as a reference region for 12 selected ROIs, i.e., amygdala, basal forebrain septum, cerebellum, cingulate cortex, hippocampus, hypothalamus, inferior colliculi, midbrain, neocortex, striatum, superior colliculi, and thalamus. Figure 2C shows the strong agreement between DVR using IV data (DVR_IV_) and using IM data (DVR_IM_), and also demonstrates how kinetic modeling properly corrects for individual differences in the input function (Figure 2A). Furthermore, IM injections are much easier to perform than IV injections, especially in mice and neonatal rats, which have small tail veins. By adopting ^18^F-labeled tracers and IM injection, we currently have the capability of conducting SV2A PET scans in up to nine mice or six rats per radiosynthesis (three sequential scans per radiosynthesis (see below), three mice or two rats per scanner (or more if more than one small animal scanner is available).

SUV ratio (SUVR) using reference regions can also increase throughput. SUVR from 30–60 min post-injection using brain stem or cerebellum as reference regions showed great correlations with DVR or *BP*_ND_ for both [^11^C]UCB-J (Toyonaga et al., 2019) and [^18^F]SynVesT-1 (Sadasivam et al., 2020). In the validation study for injection methods, SUVR with IV injection underestimated the DVR for Subject 1 (Figure 2D) due to the slower tracer uptake into the circulation system as we discussed above. In contrast, SUVR with IM injection showed an excellent correlation with DVR (Figure 2E). Overall, the IM injection is easier to perform and produced more robust quantification data than IV injections.

### Molar Activity and Injection Mass

^18^F tracers enable us to conduct multiple sequential scans which increases study throughput. One concern of multiple sequential scans would be any potential mass effect. If the same amount of radioactivity is administered, the animals scanned at later times would be administered more cold (unlabeled) drugs than those with earlier scans. The correlation between injected mass and SUV (30–60 min post-injection) from 11 mice scans conducted with [^18^F]SynVesT-1 is shown in Figure 2F. The injected mass ranged from 33 to 68 ng and there is no negative trend between the injected mass and SUV, i.e., there is no detectable mass effect. In our studies, we have produced [^18^F]SynVesT-1 47 times between January 2019 and March 2021 with a molar activity of 259.7 ± 110.6 MBq/nmol. In total, 213 [^18^F]SynVesT-1 injections were performed in mice with >95% given mass below 60 ng (Figure 2G). In conclusion, the yield of [^18^F]SynVesT-1 was high enough for multiple sequential scans.

As mentioned above, IM injection was helpful to increase the study throughput, with one limitation being the total injectable volume. The maximum injectable volume for a mouse is 0.05 ml per thigh and 0.3 mL per thigh for a rat. The final concentration of [^18^F]SynVesT-1 was high enough (injected activity:6.9 ± 3.2MBq, *n* = 48) for the limited volume of IM injection.

### Prospects

SV2A PET imaging can be used to reliably quantify SV2A changes in rodent models of neuropsychiatric disorders, track disease progression, and monitor the therapeutic effects of experimental interventions. With the optimized imaging protocol and the corresponding data analysis method properly validated, relatively high throughput animal imaging studies are feasible using ^18^F-labeled SV2A PET tracers. We expect to see the broad impact of this non-invasive and sensitive *in vivo* quantification method in basic neurological research as well as in the drug discovery and development process.

## Data Availability Statement

The original contributions presented in the study are included in the article/supplementary material, further inquiries can be directed to the corresponding author/s.

## Ethics Statement

The animal study was reviewed and approved by Yale University’s Institutional Animal Care and Use Committee.

## Author Contributions

TT and ZC directed the manuscript structure together. TT wrote the sections for imaging method and image analysis. ZC wrote the sections for tracer characteristics and applications. AF-Z and SS provided the animals for PET imaging and shared the experiences on Alzheimer’s disease and traumatic brain injury model animals for writing. RC supervised the entire manuscript. All authors contributed to the article and approved the submitted version.

## Author Disclaimer

The content is solely the responsibility of the authors and does not necessarily represent the official views of the National Institutes of Health.

## Conflict of Interest

ZC is an Archer Foundation Research Scientist. The radioligand [^18^F]SynVesT-1 is the subject of international patent application PCT/US2018/018388, “Radiolabeled Pharmaceuticals and Methods of Making and Using Same,” filed on February 15, 2018 (ZC and RC are 2 of the 5 co-inventors). The remaining authors declare that the research was conducted in the absence of any commercial or financial relationships that could be construed as a potential conflict of interest.

## Publisher’s Note

All claims expressed in this article are solely those of the authors and do not necessarily represent those of their affiliated organizations, or those of the publisher, the editors and the reviewers. Any product that may be evaluated in this article, or claim that may be made by its manufacturer, is not guaranteed or endorsed by the publisher.
